# Impact of Surgical Resection After Induction Gemcitabine Plus S-1-Based Chemoradiotherapy in Patients with Locally Advanced Pancreatic Ductal Adenocarcinoma: A Focus on UR-LA Cases

**DOI:** 10.3390/cancers17061048

**Published:** 2025-03-20

**Authors:** Masashi Kishiwada, Shugo Mizuno, Aoi Hayasaki, Benson Kaluba, Takehiro Fujii, Daisuke Noguchi, Takahiro Ito, Yusuke Iizawa, Akihiro Tanemura, Yasuhiro Murata, Naohisa Kuriyama

**Affiliations:** Department of Hepatobiliary Pancreatic and Transplant Surgery, Mie University School of Medicine, 2-174 Edobashi, Tsu 514-8507, Mie, Japan; kishiwad@med.mie-u.ac.jp (M.K.); a-hayasaki@med.mie-u.ac.jp (A.H.); kalubenson@med.mie-u.ac.jp (B.K.); t-fujii@med.mie-u.ac.jp (T.F.); nnndaisuke@med.mie-u.ac.jp (D.N.); itotakahiro@med.mie-u.ac.jp (T.I.); uskm007@med.mie-u.ac.jp (Y.I.); iorichan@med.mie-u.ac.jp (A.T.); yasumura@med.mie-u.ac.jp (Y.M.); naokun@med.mie-u.ac.jp (N.K.)

**Keywords:** chemoradiotherapy, gemcitabine, S-1, pancreatic ductal adenocarcinoma, locally advanced unresectable PDAC, performance status, CA19-9, histological response, PNI, adjuvant chemotherapy

## Abstract

**Background:** This study aimed to assess the safety and efficacy of gemcitabine plus S-1-based chemoradiotherapy (GS-CRT) among patients with locally advanced pancreatic ductal adenocarcinoma (PDAC), especially among those with unresectable locally advanced (UR-LA) cases. **Methods**: A total of 351 consecutive PDAC patients were enrolled and prognostic predictors of disease-specific survival (DSS) were identified. **Results:** The treatment completion rate was 98.9% and Grade 3 or higher adverse events occurred in 181 cases (51.6%). Among 319 re-evaluated patients, pancreatectomy was performed in 184 (57.7%). Based on resectability, the 5-year DSS rates for the entire cohort were 39.6% (R), 43.8% (BR-PV), 21.2% (BR-A) and 13.3% (UR-LA), while the predictors of DSS were performance status (PS), hemoglobin (Hb) level, celiac artery (CA) involvement of ≥180 degrees and JPS 8th T category. In the resected cases, the predictors of DSS were preoperative PS, preoperative CA19-9 level, preoperative JPS-T factor, degree of histological response and adjuvant chemotherapy. In UR-LA resected patients, preoperative prognostic nutritional index (PNI), absence of pathological venous invasion and adjuvant chemotherapy were predictors of DSS. **Conclusions:** Even though Grade 3 or higher adverse events were encountered in about half of the cases, they were uneventfully managed. Therefore, GS-CRT is safe and highly tolerable with potential to improve patients‘ prognosis. Preoperative PS, CA19-9 levels and histological response are important prognostic factors, as well as adjuvant therapy. In UR-LA patients, prognostic nutritional index (PNI) and adjuvant chemotherapy were important for curative intent surgery.

## Simple Summary

Treatment safety and efficacy of the local GS-CRT protocol were assessed in 351 patients treated at our institution for pancreatic ductal adenocarcinoma (PDAC). Prognostic predictors of survival were also identified. The GS-CRT was well tolerated as the treatment completion rate was 98.9% and the patients developed only manageable side effects. After treatment, 184 (52.4%) patients underwent surgical resection, which included 49 (26.6%) UR-LA cases in whom a 79.6% R0 resection rate was achieved. Prognostic predictors of disease-specific survival (DSS) among the resected cases included performance status (PS 0–1), CA19-9 level (<28.4 U/L), JPS-T factor (T1–3) and histological response (≥Grade 3), whereas among UR-LA resected patients, preoperative prognostic nutritional index (PNI ≥ 43.2), pathological absence of venous invasion (v0) and adjuvant chemotherapy were associated with a better prognosis. GS-CRT is a safe and effective option that improves both resection rates and survival outcomes for PDAC patients, including those with advanced (UR-LA) cases. This emphasizes the importance of comprehensive preoperative and postoperative care in improving patients’ outcomes.

## 1. Introduction

Pancreatic ductal adenocarcinoma (PDAC) is one of the most aggressive solid malignancies with a 5-year survival rate of approximately 13% [1]. Globally, it accounts for about 500,000 deaths annually [2] and, consequently, ranks as the fourth leading cause of cancer-related deaths in the United States [1] and third in Japan [3]. In both of these countries, PDAC is second only to colorectal cancer as the leading cause of gastrointestinal-cancer-related deaths [1,3]. In diagnosing PDAC, radiological imaging technologies play a very important role. Therefore, the recent advancement of integrating diagnostic images with artificial intelligence (AI) technologies has significantly improved their diagnostic accuracy. As a result, this has led to an increase in the early detection of pancreatic cancer cases [4,5,6]. Unfortunately, the majority (80–85%) of patients are initially diagnosed at an advanced stage, making surgical resection unfeasible. However, among the remaining 15–20% who present with potentially resectable disease, 30–35% have locally advanced PDAC [1]. Over the past two decades, advances in systemic therapies and pre-operative staging criteria based on resectability classifications have improved the management of PDAC cases and patients’ survival outcomes. For instance, in 2006, the National Comprehensive Cancer Network (NCCN) introduced guidelines which defined “borderline resectable (BR)” tumors as those at high risk for positive surgical margins following resection [7,8]. Since then, other organizations such as the American Hepatopancreatobiliary Association (AHPBA), the Society of Surgical Oncology (SSO) [9], the University of Texas MD Anderson Cancer Center (MDACC) [10], Alliance [11], and JPS [12,13,14] have developed their own guidelines or classification. These classifications evaluate tumor resectability based on tumor interface with such major blood vessels as the superior mesenteric vein/portal vein (SMV/PV), superior mesenteric artery (SMA) and celiac artery (CA) to predict surgical outcomes.

The management of non-metastatic PDAC often requires a multimodal approach that involves a combination of surgery, chemotherapy and radiotherapy. However, the approach slightly differs depending on the tumor resectability. Therefore, for resectable cases, the standard treatment is surgery first, followed by adjuvant chemotherapy. Among adjuvant chemotherapy regimens, mFOLFIRINOX has been shown to be the most effective as it significantly improves median overall survival (OS) compared to gemcitabine monotherapy (MST: 53.5 months vs. 35.5 months) [15]. In contrast, for borderline (BR) and unresectable–locally advanced (UR-LA) PDAC, neoadjuvant therapy is recommended in order to increase the likelihood of achieving R0 resection. FOLFIRINOX and gemcitabine/nab-paclitaxel (GnP) are considered the standard chemotherapy treatment [16,17,18,19]. In some selected patients with initially advanced disease, conversion surgery is feasible following CRT, thereby leading to improved survival outcomes [19].

Additionally, several studies have reported the significance of neoadjuvant therapy in improving survival outcomes for PDAC patients. For instance, the Prep-02/JSAP-05 trial found that resectable (R) PDAC patients who received a pre-operative combination treatment of gemcitabine and S-1 had a significantly longer median overall survival (OS) compared to those who received upfront surgery (UFS) followed by adjuvant chemotherapy; the median survival time (MST) was 36.7 vs. 26.6 months, *p* = 0.015 [20]. However, another study by the ALLIANCE A021806 trial also reported improved disease-free survival (DFS) with peri-operative mFOLFIRINOX compared to post-operative therapy alone [21]. However, other studies such as the SWOG 1505 [22] and PANACHE01-PRODIGE48 trials [23] did not demonstrate a survival benefit for preoperative chemotherapy compared to patients who had UFS.

In recent years, assessing the efficacy of pre-operative CRT has also been of significant interest in previous reports. However, these reports studied this treatment efficacy based on patients’ tumor resectability. Consequently, some focused only on either R or borderline-resectable (BR) cases, while others involved unresectable–locally advanced (UR-LA) cases. For example, the PREOPANC-1 trial compared gemcitabine-based pre-operative CRT with upfront surgery among patients with either R or BR-PDAC and demonstrated survival benefits of CRT only for BR patients [24], while the PREOPANC-2 trial compared patients ‘outcomes between the FOLFIRINOX-based and gemcitabine-based preoperative CRT. Their study observed similar resection rates, overall survival outcomes and incidences of serious adverse events between the two treatment groups [25]. In another study involving BR-PDAC cases, the ESPAC-5 trial observed that one-year survival rates were 39% in the immediate surgery group, 78% in the gemcitabine plus capecitabine group, 84% in the FOLFIRINOX group, and 60% in the capecitabine-based CRT group, which showed favorable results for the neoadjuvant group. However, even though tumor shrinkage was anticipated in the CRT group, it tended to be inferior compared to the chemotherapy-alone group based on the one-year survival and resection rates [26]. Additionally, the Alliance A021501 trial demonstrated that survival outcomes after pre-operative mFOLFIRINOX treatment alone were superior to those of combined chemotherapy and radiation therapy (MST: 29.8 vs. 17.1 months) among BR-PDAC cases [27]. However, the dose of stereotactic body radiation therapy (SBRT) used in this trial was relatively low at 25–40 Gy in five fractions and it was not administered concurrently with chemotherapy. These factors may have contributed to the lack of improvement in survival outcomes in the CRT group.

In studies which focused on UR-LA cases, the SCALOP trial included PDAC patients who either responded to or maintained stable disease after induction chemotherapy with gemcitabine plus capecitabine. These patients were randomly assigned to these two CRT regimens and the results showed improved survival (median survival time: 15.2 vs. 13.1 months) and fewer adverse events in the capecitabine-based CRT group [28], while the CONKO-007 trial investigated the efficacy of CRT with gemcitabine + radiotherapy after induction chemotherapy with gemcitabine or FOLFIRINOX compared to chemotherapy alone (gemcitabine or FOLFIRINOX). Their CRT group had significantly higher rates of pathological complete response (pCR) rate compared to the chemotherapy-only group (18% vs. 2%, *p* = 0.0043). However, no improvement in survival was observed (5-year survival rate: 9.6% vs. 4.3%, *p* = 0.713) [29]. These findings suggest that the addition of CRT does not necessarily contribute to an extension of overall survival. Lastly, patients who did not exhibit disease progression after four months of induction chemotherapy with gemcitabine or gemcitabine plus erlotinib were enrolled in the LAP07 trial. They were randomly assigned to either the CRT group, which received capecitabine plus radiotherapy (54 Gy), or the chemotherapy-alone group, which continued treatment with gemcitabine or gemcitabine plus erlotinib. Even though, the CRT group demonstrated better local disease control compared to the chemotherapy-alone group (32% vs. 46%, *p* = 0.03), no significant improvement in overall survival was observed either (MST: 15.2 months vs. 16.5 months, *p* = 0.83) [30]. This also proved that the addition of CRT did not improve overall survival in these patients with UR-LA PDAC. Consequently, the efficacy of pre-operative CRT in advanced PDAC cases remains unclear, highlighting the need for further studies to optimize treatment strategies.

Our local protocol of gemcitabine-based chemoradiotherapy (G-CRT) followed by surgery for patients with localized PDAC was launched in 2005. Consequently, we have been prospectively recruiting patients with R, BR and unresectable–locally advanced (UR-LA) tumors. Furthermore, the treatment efficacy of the protocol has previously been reported [31]. Shortly after the protocol was rolled out, several studies in Japan reported the superiority of gemcitabine + S-1 combination chemotherapy (GS-CRT) over gemcitabine alone [32,33], which, in turn, led to us switching our G-CRT to the GS-CRT protocol as the preoperative treatment in September 2011. In a comparison of 113 patients who underwent curative resection (G-CRT: 60 vs. GS-CRT: 53), Takeuchi et al. assessed short-term outcomes, which showed that the GS-CRT group had a significantly higher R0 resection rate (*p* = 0.013) and longer disease-specific survival (MST: 36.0 vs. 27.2 months, *p* = 0.042) compared to the G-CRT group. Notably, among UR-LA cases, the GS-CRT group (n = 18) demonstrated significantly improved long-term survival compared to the G-CRT group (n = 23), with MST of 36.0 vs. 18.1 months, *p* = 0.014 [34], while a recent study by Kato et al. reported that in a subgroup analysis of 72 patients with UR-LA who underwent curative resection (G-CRT: 29 vs. GS-CRT: 43), CEA was identified as an independent poor prognostic factor at a cutoff value of 7.2 ng/mL. Therefore, patients with high CEA levels (n = 15) had significantly poor survival rates compared to those with low CEA levels (n = 57) with MST of 8.0 vs. 24.0 months, *p* < 0.001). They also concluded that the CEA level at diagnosis prior to CRT was an important prognostic indicator for UR-LA PDAC [35]. Furthermore, among 203 patients who underwent curative resection (G-CRT: 64 vs. GS-CRT: 139), Murata et al. demonstrated that the GS-CRT group had a significantly higher proportion of high responders, which was defined as a histologic response of 90% or greater, compared to the G-CRT group (31.7% vs. 6.3%, *p* < 0.01). Additionally, the 5-year survival rate was significantly prolonged in the GS-CRT group (36.4% vs. 23.4%, *p* = 0.04) [36].

In the current study, we assessed the impact of surgical resection after induction GS-CRT and examined adverse events associated with preoperative treatment as well as long-term outcomes based on resectability category. This study also aimed to identify prognostic predictors of survival across the diagnostic, perioperative and postoperative periods, with a particular focus on UR-LA cases.

## 2. Materials and Methods

### 2.1. Study Design

**Figure 1:**
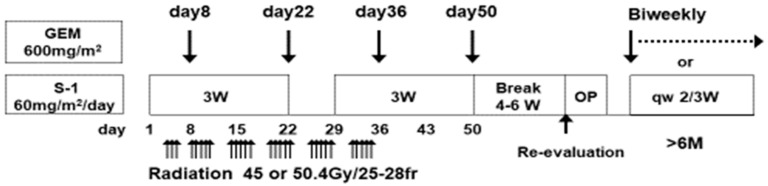
Gemcitabine plus S-1-based chemoradiotherapy (GS-CRT) protocol at Mie University Hospital. GS-CRT (2011.9–2021.8, enrolled patients: n = 351, re-evaluated patients: n = 319) GEM: Gemcitabine, W: Week, OP: Operation, m: Months, qw: Every week.

From September 2011 to August 2021, we enrolled 351 patients who were diagnosed with localized PDAC through either cytological or histological specimens obtained from endoscopic ultrasound-guided fine-needle aspiration (EUS-FNA). During this period, a GS-CRT treatment protocol was administered to these patients (Figure 1). However, patients who were identified to have metastatic disease were not included.

**Figure 2:**
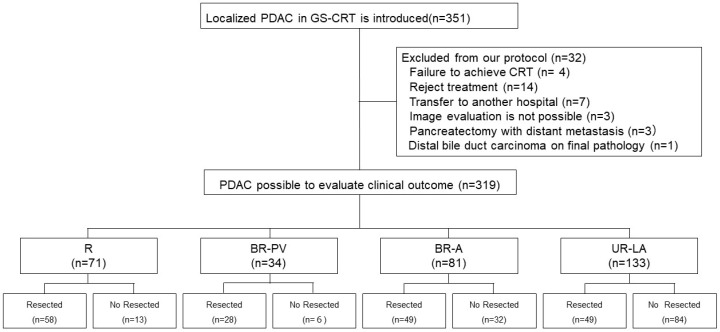
Flow diagram of the 351 PDAC patients who were enrolled in the GS- CRT protocol. Among 351 enrolled in GS-CRT, 319 were re-evaluated for clinical outcomes (R: 71, BR-PV: 34, BR-A: 81, UR-LA: 133). PDAC: Pancreatic ductal adenocarcinoma, CRT: Chemoradiotherapy, R: Resectable, BR-PV: Borderline resectable (superior mesenteric vein/portal vein invasion alone), BR-A: Borderline resectable (arterial invasion), UR-LA: Locally advanced.

Based on tumor resectability classified according to the JPS 8th resectability classification guidelines [12,13,14], all patients were grouped into the R, BR-PV, BR-A, and UR-LA categories. Tumor resectability was evaluated on the patient’s first visit to our hospital based on anatomical definitions using 64 slice MDCT imaging which included both coronal and sagittal views. However, among the 351 initially patients enrolled in the GS-CRT protocol, 319 were re-evaluated for clinical outcomes and categorized as follows: R (n = 71), BR-PV (n = 34), BR-A (n = 81), and UR-LA (n = 133) (Figure 2).

All patients provided written informed consent for inclusion in this study. The study protocol was approved by the Medical Ethics Committee of Mie University Hospital (Approval No. H2020-118) and was conducted in accordance with the ethical standards of the latest version of the Declaration of Helsinki which was revised in 2024.

### 2.2. Treatment Protocol

All the patients received S-1 orally twice daily at a dose of 60 mg/m^2^ on days 1 to 21 in a 28-day cycle. They also received a gemcitabine infusion at a dose of 600 mg/m^2^ on days 8, 22, 36, and 50, respectively. As previously described, S-1 is an oral agent containing tegafur, gimeracil, and oteracil [34,35,36,37,38,39]. The patients concurrently underwent 3D conformal radiotherapy as previously reported [31,34,35,36,37,38,39]. Radiotherapy was initiated within 14 days after the start of chemotherapy and was administered to a total dose of 50.4 Gy (1.8 Gy per day, five times per week, for a total of 28 fractions). The radiation field was set using an MDCT with a slice thickness of 2.5 mm. The clinical target volume (CTV) included the gross tumor volume of the primary pancreatic tumor and clinically diagnosed lymph node metastases with a 5 to 10 mm margin. The CTV also generally encompassed a 10 mm margin around the SMA and CA. The planning target volume was defined as the CTV with an additional margin to account for organ motion due to respiration and setup errors. A 10-MV linear accelerator was used for dose delivery, with careful attention paid to avoid excessive radiation exposure to the spinal cord (<50 Gy) and kidneys (mean dose < 18 Gy). Lastly, adverse events during induction GS-CRT were assessed using the CTCAE v5.0.

### 2.3. Indications of Surgical Resection and Post-Operative Complications

#### 2.3.1. Indications and Contraindications of Surgical Resection

All the patients underwent re-assessment 4 to 6 weeks after CRT. The indications for surgical resection were determined based on a comprehensive assessment of tumor shrinkage, presence or absence of distant metastasis, tumor marker trends, and the patient’s overall condition. When curative-intent resection was deemed feasible, they were scheduled for pancreatectomy, especially those found to have R and BR-PV tumors. For cases initially classified as BR-A or UR-LA, curative-intent resection was only considered feasible if tumor down-staging from BR-A/UR-LA to R/BR-PV/BR-A was observed at the time of post-CRT re-evaluation. Nevertheless, surgical resection was also considered for those without tumor down-staging if there was no encasement or deformity of the celiac artery (CA), superior mesenteric artery (SMA) or jejunal arteries detected on dynamic CT-scan images. Other indications for surgery included absence of distant metastases, normalization or a significant decrease in post-CRT serum CA 19-9 compared to the pre-CRT levels, and a performance status (PS) of 0–1.

For patients with advanced PDAC, previously reported clinical data by the Japanese Society of Hepato-Biliary-Pancreatic Surgery showed favorable overall survival (OS) rates for UR-LA patients who received initial CRT treatment, especially those who received non-surgical anti-cancer treatment for 240 days or more from start of treatment [40]. As a result, there has been a push towards administering additional chemotherapy following CRT since 2013 in these advanced cases. Finally, for patients who did not satisfy the criteria for pancreatectomy, they continued to receive GS chemotherapy and were re-evaluated by MDCT after two additional cycles. Pancreatectomy was only planned if re-evaluation indicated suitability for surgical resection.

#### 2.3.2. Surgical Procedure

Following re-evaluation, either pancreaticoduodenectomy (PD), distal pancreatectomy (DP) or total pancreatectomy (TP) was performed depending on tumor location (head, body or tail). SMV/PV resection and subsequent vascular reconstruction were conducted if the surgeon was unable to dissect off the pancreatic head from these vessels without leaving gross tumor on the vessels. Segmental resection and re-anastomosis of the common hepatic artery (CHA) was also performed if tumor involvement was limited to the CHA. Regarding lymph node dissection, no extended lymph node dissection was performed except for regional lymph node dissection as defined by the JPS 8th classification [12,13,14]. Intra-operatively, those patients who were found to be unresectable, usually due to the presence of distant metastases, underwent surgical bypass according to clinical indications.

#### 2.3.3. Post-Operative Complications

Postoperative complications, which included morbidity and mortality, were assessed based on the Clavien–Dindo classification [41]. Post-operative mortality was defined as the 90-day in-hospital mortality.

### 2.4. Adjuvant Chemotherapy and Post-Operative Follow-Up

Following surgical resection, adjuvant chemotherapy with gemcitabine or S-1 was initiated within 6 weeks of surgery and was planned to continue for at least 6 months, as previously reported [34,35,36,37,38,39]. All patients were followed up in an outpatient setting where monthly physical examinations were performed and blood tests which included serum levels of CA19-9 were carried out every 2–3 months. Additionally, MDCT was conducted every 3–6 months within the first 2 years and every 6 months thereafter. Recurrence sites were recorded at the time of the first recurrence.

### 2.5. Pathological Evaluation of Histological Response to Chemoradiotherapy

The resected specimens were fixed in formalin solution, and then sectioned at approximately 5 mm intervals and embedded in paraffin blocks. A 3 μm section was taken from each block and stained with hematoxylin–eosin. The sections were then evaluated for pathological differentiation, lymph node metastasis, lymphatic invasion, venous invasion, perineural/neural invasion, extension to the main pancreatic duct, and residual tumor (R) status. Resection margin (R0/R1) status was evaluated according to the histological criteria of the JPS classification [12,13,14]. R1 was defined as a microscopically incomplete resection with 0 mm clearance, while R0 indicates no residual tumor on surgical specimens. Histological assessment of the pre-operative response to CRT was also carried out according to the JPS classification. The residual tumor rate was defined as the ratio of the volume of viable cancer cells to the estimated tumor volume before treatment. Furthermore, if determining the grade was challenging, the lower effectiveness grade was selected [12,13,14].

### 2.6. Statistical Analyses

Continuous variables were expressed as medians (range). The initial treatment date was set as the starting point for survival time measurement among all the re-assessed patients. Consequently, disease-specific survival (DSS) was calculated in months from the time of disease diagnosis, and patients who were alive or had died from causes other than PDAC were censored for the analysis of DSS. DSS was calculated using the Kaplan–Meier method and survival rates were compared using the log-rank tests. Patients were recruited over a 10-year period (from September 2011 to August 2021) and the final case was enrolled on 23 August 2021. For the follow-up review, medical records were examined up to 31 August 2024. For the surviving patients who had not visited the outpatient clinic for more than six months, their status was confirmed via telephone and no patient was lost to follow-up. The median follow-up duration after initial treatment was 22.0 months (range: 3.4–155.0 months). Risk factors associated with grade 3 or higher adverse events were analyzed using the Mann–Whitney U and chi-square tests. Statistical analyses were conducted using SPSS version 29 (SPSS Inc., Chicago, IL, USA), with a *p*-value of less than 0.05 considered statistically significant.

## 3. Results

### 3.1. Adverse Events During Induction GS-CRT

Among the 351 recruited patients, 347 successfully completed the induction GS-CRT, resulting in a high completion rate of 98.9%. Based on tumor resectability, the completion rates were 96.4% (R), 97.5% (BR-PV), 100% (BR-A) and 100% (UR-LA). Therefore, only four patients discontinued treatment. Unfortunately, grade 3 or higher adverse events were observed in 181 cases (51.6%) with hematological toxicities being the commonest. Specifically, 139 patients (39.6%) developed leukopenia, and 113 patients (32.2%) had neutropenia, while febrile neutropenia was reported in only two patients (0.6%). Gastrointestinal symptoms were rare, with only 13 patients (3.7%) experiencing anorexia, and one patient (0.3%) had diarrhea. Most importantly, no cases of eczema or peripheral neuropathy were reported. Furthermore, no treatment-related deaths occurred during the induction phase (Table 1).

### 3.2. Characteristics of Patients with Localized Pancreatic Ductal Adenocarcinoma

Table 2 shows the clinical characteristics of the 319 patients who were enrolled for re-assessment at the time of PDAC diagnosis. Among them, 95.6% (305/319) had a PS of 0–1 and the median values of the key blood tests were 13.1 g/L (Hb), 38.5 (PNI), 199.4 U/L (CA19-9) and 3.7 ng/mL (CEA), respectively. On dynamic CT scan images, the median tumor size was 32.9 mm and the incidence of tumor–vascular contact or invasion of ≥180° was 47.0% (150/319) for SMV/PV, 17.2% (55/319) for CA, 24.1% (77/319) for SMA, and 18.1% (67/319) for CHA contact.

However, among the 184 resected cases (Table 2), pre-operative factors included PS 0–1 in 97.8% (180/184) with median blood laboratory values of 11.5 g/L (Hb), 41.0 (PNI), 28.9 U/L (CA19-9) and 3.0 ng/mL (CEA). The median tumor was 25.7 mm and the incidence of tumor–vascular invasion or contact of ≥180° was 36.4% (67/184) for SMV/PV, 9.2% (17/184) for CA, 15.2% (28/184) for SMA and 15.2% (28/184) for CHA. Intra-operatively, PD or TP were performed in 82.6% (152/184), while 32 (17.4%) underwent DP with a median operative time of 525 min and blood loss of 757 mL. Concomitant SMV/PV resection and subsequent vascular reconstruction was performed in 76.6% (141/184), CHA resection in 9.8% (18/184), and CA resection in 3.3% (6/184) patients, respectively. Regarding pathological outcomes, 8.6% (158/184) had no lymphatic invasion (ly0), 39.1% (72/184) had no perineural invasion (ne0), and 79.9% (147/184) had no venous invasion (v0). The R0 resection rate was 91.3% (168/184) and 37.0% (68/184) of cases had a histological effect of 90% or more. In the post-operative period, grade 3 or higher postoperative complications occurred in 28.9% (53/184) of the cases and adjuvant therapy was administered in 84.2% (155/184) of patients.

For the 49 resected cases with UR-LA tumors (Table 2), 98.0% (48/49) had a preoperative PS of 0–1 and the median blood laboratory values were 11.2 g/L (Hb), 39.3 (PNI), 27.4 U/L (CA19-9) and 2.8 ng/mL (CEA). Similarly, the median tumor size was 29.7 mm and tumor–vascular interface (contact or invasion) of ≥180° was 57.1% (28/49) for SMV/PV, 30.6% (15/49) for CA, 46.9% (23/49) for SMA and 44.9% (22/49) for CHA. Pancreaticoduodenectomy or TP were performed in 79.6% (36/49), while the remaining 13 patients (20.4%) had DP with a median operative time of 600 min and blood loss of 1155 mL. In these patients with advanced disease, combined SMV/PV resection and vascular reconstruction was performed in 79.6% (39/49), CHA resection in 20.4% (10/49), and CA resection in 10.2% (5/49). For pathological characteristics, ly0 was observed in 87.8% (43/49), ne0 in 22.4% (11/49) and v0 in 85.7% (42/49). The R0 resection rate was 79.6% (39/49) and a histological effectiveness of 90% or more was seen in 73.5% (36/49) of the cases. Postoperative factors showed that grade 3 or higher post-operative complications occurred in 26.5% (13/49) of cases. Lastly, 79.6% (39/49) of patients received adjuvant therapy.

### 3.3. Timing of Surgical Resection Based on Tumor Resectability Classification

**Figure 3:**
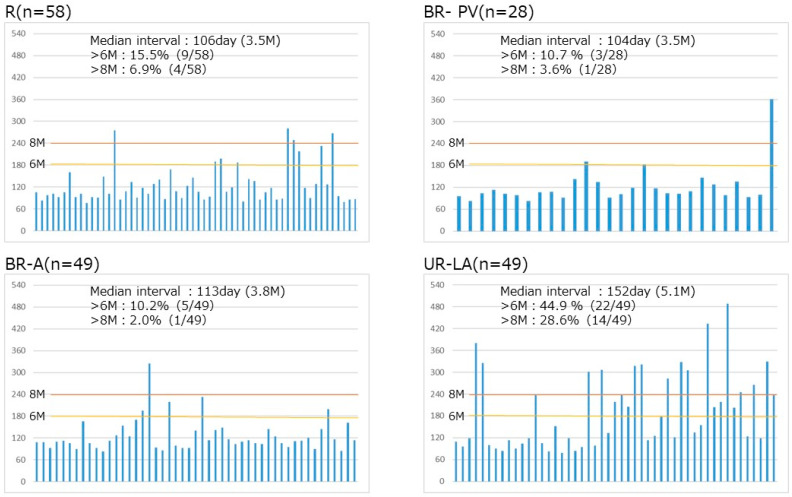
Interval from initial treatment to surgical resection based on tumor resectability (R: 58, BR-PV: 28, BR-A: 49, UR-LA: 49). Median intervals (days) to surgery were R: 106, BR-PV: 104, BR-A: 113, UR-LA: 152. In addition, 10.9% (20/184) of the cases underwent resection after more than 8 months of initial treatment. Based on tumor resectability, these were 6.9% (4/58) R, 3.6% (1/28) BR-PV, 2.0% (1/49) BR-A and 28.6% (14/49) UR-LA cases, respectively. R: Resectable, BR-PV: Borderline resectable (superior mesenteric vein/portal vein invasion alone), BR-A: Borderline resectable (arterial invasion), UR-LA: Locally advanced.

The median duration from initiation of initial treatment to surgery for all the resected cases (n = 184) was 105 days. However, surgery was delayed for ≥6 months in 21.2% and for ≥8 months in 10.9% of cases, respectively. Based on tumor resectability, the median intervals from treatment to surgery were similar: 106 (R), 104 (BR-PV) and 113 (BR-A) days, respectively. Additionally, they all had low rates of cases with delayed resection for both ≥6 months and ≥8 months. In contrast, UR-LA cases had a longer median interval from treatment to surgery of 152 days with significantly higher rates of delayed resection cases: 44.9% (≥6 months) and 28.9% (≥8 months), respectively (Figure 3).

### 3.4. Survival Analyses Based on Tumor Resectability

**Figure 4:**
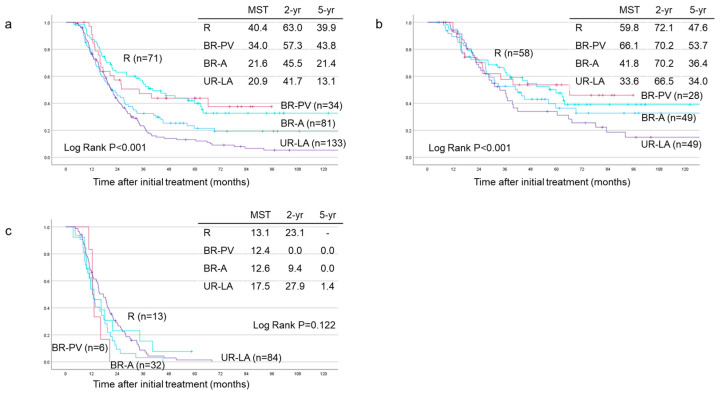
Disease-specific survival (DSS) curves of GS-CRT PDAC patients based on tumor resectability according to JPS classification. (**a**) All patients (n = 319, R: 71, BR-PV: 34, BR-A: 81, UR-LA: 133), (**b**) resected patients (n = 184, R: 58, BR-PV: 28, BR-A: 49, UR-LA: 49), and (**c**) unresected patients (n = 135, R: 13, BR-PV: 6, BR-A: 32, UR-LA: 84). JPS 8th: The eighth edition of General Rules for the Study of Pancreatic Cancer published by the Japan Pancreas Society. R: Resectable, BR-PV: Borderline resectable (superior mesenteric vein/portal vein invasion alone), BR-A: Borderline resectable (arterial invasion), UR-LA: Locally advanced, MST: Median survival time (months).

Based on tumor resectability classified according to the JPS classification among the 319 re-evaluated patients, the MST, 2-year and 5-year survival rates were significantly different across the groups (*p* < 0.001) (Figure 4a). The MST, 2-year and 5-year survival rates were R (n = 71), 40.4 months, 63.0%, and 39.9%; BR-PV (n = 34), 34.0 months, 57.3%, and 43.8%; BR-A (n = 81), 21.6 months, 45.5%, and 21.4%; and UR-LA (n = 133), 20.9 months, 41.7%, and 13.1%, respectively. For patients who underwent pancreatectomy, significant differences in MST, 2-year and 5-year DSS rates were observed (*p* < 0.001): R (n = 58), 59.8 months, 72.1%, and 47.6%; BR-PV (n = 28), 66.1 months, 70.2%, and 53.7%; BR-A (n = 49), 41.8 months, 70.2%, and 36.4%; and UR-LA (n = 49), 33.6 months, 66.5%, and 34.0%, respectively (Figure 4b). However, in the unresected cases, the MST, 2-year and 5-year survival rates did not differ significantly among the groups (*p* = 0.122) (Figure 4c): R (n = 13), 13.1 months, 23.1%, and not available (NA); BR-PV (n = 6), 12.4 months, 0.0%, and 0.0%; BR-A (n = 32), 12.6 months, 9.4%, and 0.0%; and UR-LA (n = 84), 17.5 months, 27.9%, and 1.4%, respectively.

### 3.5. Identifying Significant Prognostic Predictors of Disease-Specific Survival

#### 3.5.1. Among the 319 Re-Evaluated Cases

Among the 319 re-evaluated PDAC cases, multivariate analysis identified PS 0–1 (*p* = 0.04), high initial Hb level (*p* = 0.006), CA contact or invasion < 180° (*p* = 0.029) and JPS 8th T1–3 factor (*p* < 0.001) as independent prognostic predictors for favorable DSS, as shown in Table 3. Supplementary Figure S1 illustrates the impact of PS and Hb levels on DSS. Unfortunately, DSS was not significantly different between patients with PS 0–1 (n = 305) and PS 2–3 (n = 14), *p* = 0.069. However, patients with Hb ≥ 13.5 (n = 135) had a significantly longer MST compared to those with Hb < 13.5 (n = 182) (31.3 vs. 21.4 months, *p* = 0.007).

#### 3.5.2. Among the 184 Patients Who Underwent Surgical Resection

**Figure 5:**
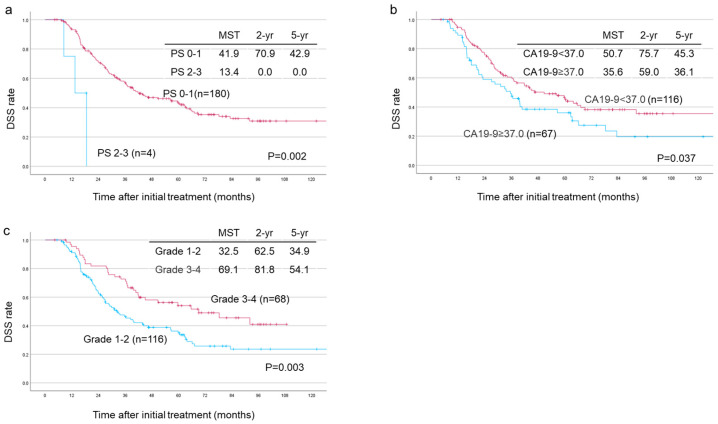
Disease-specific survival (DSS) curves of resected patients (n = 184). (**a**) MST in patients with PS 0–1 (n = 180) was significantly longer than that in patients with PS 2–3 (n = 4) (MST 41.9M vs. 13.4, *p* = 0.002). (**b**) MST in patients with CA19-9 < 37.0 (n = 116) was significantly longer than that in patients with CA19-9 ≥ 37.0 (n = 67) (MST 50.7M vs. 35.6, *p* = 0.037). PS: Performance status, MST: Median survival time. (**c**) MST in patients with histological grade 1–2 (n = 116) was significantly lower than that in patients with grade 3–4 (n = 68) (MST 32.5M vs. 69.1, *p* = 0.003).

Similarly, among all the resected patients, multivariate analysis identified PS 0–1 (*p* = 0.006), low CA19-9 levels (*p* < 0.001), JPS 8th T1–3 factor (*p* < 0.001), grade 3–4 (histological response ≥ 90%) (*p* = 0.021) and administration of adjuvant chemotherapy (*p* = 0.006) as independent prognostic predictors of DSS, Table 4. Survival analyses based on PS showed significant differences between the PS 0–1 (n = 180) and the PS 2–3 (n = 4) groups, with MST, 2-year and 5-year survival rates of 41.9 vs. 13.4 months, 70.9% vs. 0%, and 42.9% vs. 0%, respectively, *p* = 0.002 (Figure 5a).

Since CA19-9 is the most useful tumor marker for predicting patient prognosis, an optimal cutoff value of 28.4 (*p* = 0.012) was determined using a free online Cutoff Finder software [42]. However, the normal CA19-9 value of 37 was preferred to be used as a cutoff as it is more convenient for practical use in clinical settings. Consequently, with a cutoff set at 37, further analyses were conducted by appropriately dividing the patients into groups based on CA19-9 being less and greater than 37. As expected, survival outcomes were significantly different between the two groups. The CA19-9 < 37 (n = 116) had superior outcomes compared to the CA19-9 ≥ 37 (n = 67) group with MST, 2-year and 5-year survival rates of 50.7 vs. 35.6 months, 75.7% vs. 59.0%, and 45.3% vs. 36.1%, respectively, *p* = 0.037 (Figure 5b). Similarly, a significant difference was observed between grade 3–4 (histological response ≥ 90%; n = 68) and grade 1–2 (histological response < 90%; n = 116) with MST, 2-year and 5-year survival rates of 69.1 vs. 32.5 months, 81.8% vs. 62.5%, and 54.1% vs. 34.9%, respectively, *p* = 0.003 (Figure 5c).

#### 3.5.3. Among the 49 Patients with Unresectable–Locally Advanced Tumors Who Underwent Surgical Resection

**Figure 6:**
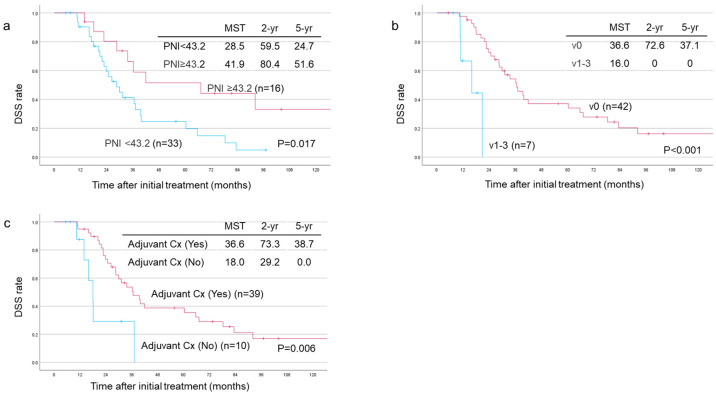
Disease-specific survival (DSS) curves of UR-LA resected patients (n = 49). (**a**) MST in patients with PNI < 43.2 (n = 33) was significantly lower than that in patients with PNI ≥ 43.2 (n = 16) (MST 28.5M vs. 41.9, *p* = 0.017). (**b**) MST in patients with pathological v0 (n = 42) was significantly longer than that in patients with v1–3 (n = 7) (MST 36.6M vs. 116.0, *p* < 0.0021). (**c**) MST in patients with Adjuvant Cx (n = 39) was significantly longer than that in patients without adjuvant Cx (n = 10) (MST 36.6M vs. 18.0, *p* = 0.006). UR-LA: Locally advanced, PNI: Prognostic nutritional index, MST: Median survival time, Cx: Chemotherapy.

In patients with UR-LA tumors, multivariate analysis revealed that high PNI (*p* = 0.02), absence of venous invasion in histopathological specimens (*p* = 0.002) and administration of adjuvant chemotherapy (*p* = 0.04) were independent prognostic predictors of favorable DSS, as shown in Table 5. The optimal pre-operative PNI cutoff value was determined to be 43.2. Additionally, survival analyses based on PNI showed a significant difference between patients with a PNI of 43.2 or greater (n = 16) and those with a PNI of less than 43.2 (n = 33) with MST, 2-year and 5-year survival rates of 41.9 vs. 28.5 months, 80.4% vs. 59.5%, and 51.6% vs. 24.7%, respectively, *p* = 0.037 (Figure 6a). For venous invasion, a significant difference was observed between the v0 (n = 42) and the v1–3 (n = 7) groups with MST, 2-year and 5-year survival rates of 36.6 vs. 16.0 months, 72.6% vs. 0%, and 37.1% vs. 0%, respectively, *p* < 0.001 (Figure 6b). Regarding adjuvant therapy, a significant difference was also found between the adjuvant therapy (n = 39) and the no-adjuvant-therapy (n = 10) groups with MST, 2-year and 5-year survival rates of 36.6 vs. 18.0 months, 73.3% vs. 29.2%, and 38.7% vs. 0%, respectively, *p* = 0.006 (Figure 6c).

## 4. Discussion

This single-center retrospective study aimed to assess the safety and efficacy of GS-CRT (Figure 1) as a pre-operative treatment modality. Additionally, its impact on survival outcomes based on tumor resectability was also evaluated. Among adverse events related to GS-CRT, myelotoxicity was the predominant one, with overall incidence rates for grade 3 or higher leukopenia and neutropenia of 39.6% and 32.2%, respectively. Nevertheless, most of these adverse events were manageable. The incidence rates of other adverse events were low and ranged from 0% to 5.1%. For example, febrile neutropenia occurred only in two patients (0.6%) and, unfortunately, one patient discontinued CRT due to these side effects of treatment, Table 1. Analysis of risk factors associated with grade 3 or higher adverse events revealed that pre-CRT white blood cell (WBC) count (*p* < 0.001) and pre-CRT neutrophil count (*p* < 0.001) were significantly associated with higher complication rates. These complications were similar based on pre-CRT resectability classification, as shown in Supplementary Table S1. As shown in Table 1, adverse events developed in 48.2% (40/83) of R, 50.0% (42/84) of BR-A, 40.0% (16/40) of BR-PV and 59.0% (85/144) of UR-LA cases. Most importantly, there were no cases of lost surgical opportunities due to toxicity. Although the use of strong regimens in preoperative treatment is ideal, avoiding lost surgical opportunities due to associated toxicities is crucial. For instance, in the SWOG clinical trial, which compared the efficacy and safety of FOLFIRINOX versus GnP as preoperative therapies in 102 eligible patients with either R or BR-PDAC, nine (approximately 9%) were unable to undergo planned surgery due to treatment-related toxicity [22]. One reason for the high tolerability in the current study was the use of a regimen which had a one-step dose reduction in S-1. In the JASPAC04 study of patients with BR-PDAC, the S1-CRT and GS-NAC regimens also employed a single-dose reduction in S-1. Their rates of grade 3 or higher leukopenia, neutropenia and febrile neutropenia were 24.0%, 59.0% and 8%, respectively, for pre-operative GS-NAC and 6.0%, 0.0% and 0%, respectively, for pre-operative S1-CRT. Additionally, they did not report any case of serious adverse events or lost surgical opportunities with these regimens [43]. Similarly, the GS-CRT protocol in the current study employed a standardized one-step dose reduction in S-1 which was considered key in preventing treatment discontinuation due to toxicity.

In the current study, cases with protocol deviations were excluded and only 319 of the 351 enrolled patients were finally included. After treatment re-evaluation, 184 patients were deemed suitable candidates and underwent surgery with resection rates of 81.7% (R), 82.3% (BR-PV), BR-A 60.5% (BR-A) and 36.8% (UR-LA), as shown in Figure 2. Similarly, R0 resection rates were 98.3% (R), 92.9% (BR-PV), 93.9% (BR-A) and 79.6% (UR-LA) based on tumor resectability. Although a high R0 resection rate was achieved in this study, many previous reports indicate that R0 resection rates are improved by concurrent radiotherapy. For instance, the JASPAC04 study reported a R0 resection rate of 97.6% (40/41) in the S1-CRT group among R-PDAC patients [44]. Similarly, the JASPAC05 study reported a R0 resection rate of 93.1% (27/29) in the S1-RT group among BR-PDAC cases [43]. Furthermore, the CONKO-007 study also observed that the R0 resection rate was significantly higher in the CRT-following-induction-chemotherapy group compared to the chemotherapy-alone group (68.6% vs. 50%; *p* = 0.0418) among advanced cases of UR-LA PDAC [29]. Even though higher R0 resection rates have also been reported in previous studies [29,43,44], similar to our findings, it is important to consider the potential influence of various biases, such as patient selection bias, responder bias, imaging-based bias, stringent surgical criteria and survivor bias. In the current study, these potential biases were not considered and this is one of this study’s limitations.

Additionally, the duration and timing of pre-operative therapy are important for improving outcomes in patients with PDAC. For us, the proportion of patients that underwent surgery at 6 months ranged from 10.2% to 15.5% and 2.0% to 6.9% at 8 months among the R, BR-PV and BR-A cases, respectively. This long interval from treatment initiation to surgery may be because many patients did not undergo resection immediately after CRT due to oncological or physical factors, such as high levels of serum CA19-9 and other tumor markers at re-assessment, poor nutritional status and poor PS, leading to delayed surgical resection after continued chemotherapy. In contrast, a higher proportion of patients with UR-LA underwent surgery at 6 months (44.9%) and 8 months (28.6%) compared to the other resectability groups, as shown in Figure 3. Among patients with initial advanced tumors (UR-LA), only three (6.2%) cases down-staged to either BR-PV (n = 2) or BR-A (n = 1). As for the R and BR cases, the timing of surgical resection was based on a comprehensive consideration of both oncological and physical factors. A study by Kim et al. suggested that 4–6 months of non-surgical therapy combined with pancreatectomy was ideal to optimize OS in R-PDAC. The authors also emphasized the need for complete neoadjuvant therapy considering that surgery can make post-operative therapy uncertain [45], while another study by Satoi et al. reported that neoadjuvant treatment of initially unresectable PDAC (UR-LA and UR-M) resulted in significantly better OS in patients treated with non-surgical anti-cancer drugs for more than 240 days [40]. Even though the NCCN pancreatic cancer guidelines recommend re-staging after the completion of neoadjuvant therapy to assess tumor response and determine the indication for surgery [7], there is still no clear evidence regarding the optimal timing of surgical resection in patients who have received chemotherapy ± radiotherapy as preoperative treatment. In view of the reasons above, the timing of surgery should be based on a comprehensive evaluation by a multidisciplinary team which takes into account the response to treatment and the patient’s general condition.

Furthermore, survival analyses were performed for all enrolled and resected cases based on tumor resectability, as shown in Figure 4a–c. Survival outcomes are usually better R compared to the BR-PV PDAC cases. However, among resected patients, we observed that the BR-PV (MST; 66.1 months) cases had a slightly longer survival than the R (MST; 59.8 months) patients. The reason for this remains unclear, but the small sample size of the BR-PV group (28 cases) could have influenced the survival outcomes, as shown in Figure 4b. In contrast, among the non-resected cases, the survival curves for all resectability categories nearly overlapped even though MSTs were slightly different among the groups (13.1 (R), 12.4 (BR-PV), 12.6 (BR-A) and 17.5 (UR-LA) months, respectively, as shown in Figure 4c). As a limitation, the current study fails to explain why survival outcomes were similar among the non-resected cases as no other analyses were conducted apart from survival ones. Based on resectability, the non-resection rates were 18.3% (R), 17.7% (BR-PV), 39.5% (BR-A) and 63.2% (UR-LA), respectively. In these patients, radiological imaging and tumor marker changes during or after pre-operative treatment may have detected subclinical distant metastases at an early stage and allowed for better monitoring of the response to pre-operative treatment, which, in turn, might have enabled better patient selection for surgical intervention. As a result, unnecessary invasive surgery can be avoided, enabling a smooth transition to the next treatment strategy while increasing treatment options and preserving the patient’s quality of life.

In multivariate analysis among the 319 re-evaluated patients, CA contact or invasion < 180° (*p* = 0.029), JPS T1–3 factor (*p* < 0.001), PS 0–1 (*p* = 0.04), and a higher initial Hb level (*p* = 0.006) were identified as prognostic predictors of favorable DSS, as shown in Table 3. In the UICC 8th edition [46], AJCC 8th edition [47] and JPS classification [12,13,14] guidelines, “CA contact or 180 degrees of invasion” and “JPS T1–3 factors (tumor diameter and extrapancreatic extension)” are used as anatomical evaluations to determine tumor stage [12,13,14]. These factors are also used as criteria to define resectability in many resectability classifications [7,9,10,11,12,13,14]. Therefore, it is reasonable that these two factors were prognostically relevant in the present study. In terms of PS, an ECOG PS of 2 or higher in the IAP criteria [7] and a PS of 3 or higher in the MDACC criteria [10] are considered conditional factors for BR-PDAC, which play a crucial role in determining both treatment strategies and patient management. A study by Tas et al. analyzed 335 patients with PDAC of all stages and found that poor PS at diagnosis (ECOG PS 2–4) was significantly associated with shorter survival outcomes in localized, locally advanced and metastatic cases [48]. The observation that the Hb level remained a prognostic factor at a cut-off value of 13.5 is particularly interesting. The Hb level is an indicator of anemia and oxygen-carrying capacity. Unfortunately, there is a limited number of studies which link Hb levels to prognosis in PDAC patients. On the other hand, several attempts to assess the overall balance of inflammation and nutritional status by combining the hemoglobin-to-red blood cell distribution width ratio (HRR) have been reported in previous studies [49,50]. For instance, Zhou et al. evaluated the HRR in 128 patients with PDAC. Their results showed that patients with a low HRR had shorter disease-free survival (DFS) and OS. These findings suggest the potential of HRR as a prognostic biomarker in PDAC patients and emphasize the importance of monitoring Hb levels [49].

Significant prognostic predictors of DSS among the 184 resected patients included preoperative PS, CA19-9 levels, JPS-T factors, histological response and adjuvant chemotherapy, as shown in Table 4. Among these, CA19-9 levels and histological response have drawn a lot of interest in several studies. However, the optimal CA19-9 cutoff value at diagnosis or the level at which benefits from neoadjuvant therapy can be expected has not yet been clearly defined. Notwithstanding, many useful reports on CA19-9 exist. For example, the IAP recommends a CA19-9 cutoff value of 500 U/mL for R-PDAC at the time of diagnosis [8,51]. Additionally, in our previous report, this cutoff value (500 U/mL) also showed differences in outcomes among R-PDAC cases [38]. Regarding the CA19-9 levels after pre-operative treatment, the NEO-LAP study focused on CA19-9 levels following multi-agent induction chemotherapy for locally advanced PDAC. In that study, while CA19-9 levels at diagnosis were not a prognostic factor for survival, CA19-9 levels after treatment were significant and <50 µ/mL was identified as the optimal cutoff [52]. In another study, Heger et al. reported that a lower pre-to-post-treatment ratio and a lower post-treatment CA19-9 level were associated with a higher chance of surgical resection, especially when the post-treatment CA19-9 level was lower than 91.8 µ/mL [53]. Additionally, Boone et al. also found that lower CA19-9 levels after CRT were associated with improved resectability and survival. Furthermore, patients with normalized CA19-9 levels after CRT had significantly better prognoses than those without normalization [54]. In a study on gemcitabine-based CRT for R and BR-PDAC, Takahashi et al. observed that R-PDAC cases with CA19-9 levels exceeding 120 µ/mL at diagnosis had worse biological prognoses than BR-PDAC cases. However, they also found that R-PDAC cases with CA19-9 levels exceeding 120 µ/mL at diagnosis had similar survival rates compared to those with CA19-9 levels lower than 120 µ/mL at diagnosis if the CA19-9 levels were reduced to less than 37 µ/mL after CRT. The authors, therefore, noted that this represents “biological down-staging” of CA19-9 from diagnosis to post-treatment [55]. As described above, CA19-9 is an important indicator for assessing the resectability and prognosis of PDAC at diagnosis and after preoperative treatment. Therefore, changes in its value are extremely useful for evaluating treatment efficacy and determining the indication for surgery.

Following pre-operative CRT, assessing histological response to pre-treatment is of great clinical importance and, consequently, several previous studies have reported the association histological efficacy and patients’ prognosis after CRT [39,56]. In our previous report on 203 PDAC patients who received either G-CRT (n = 60) or GS-CRT (n = 143), patients who achieved grade 4 (pCR) or grade 3 (residual tumor cells < 10%) histological effect had a high R0 resection rate and improved survival outcomes. Compared to G-CRT, the GS-CRT group had significantly more cases with grade 3 or higher histological effects (31.7% vs. 6.3%, *p* < 0.01). Furthermore, the 5-year survival rate was also significantly higher in the GS-CRT group (36.4% vs. 23.4%, *p* = 0.04) [39]. In the current study, which was limited to the GS-CRT group, we observed similar findings. One possible reason for the high histological efficacy and favorable prognosis of GS-CRT is the synergistic effect of combination therapy with gemcitabine (GEM) and S-1. The effectiveness of gemcitabine is significantly influenced by tumor heterogeneity and the tumor microenvironment (TME). In particular, genetic heterogeneity, such as decreased hENT1 expression and the presence of cancer stem cells (CSCs), as well as TME factors, including fibrosis induced by cancer-associated fibroblasts (CAFs) and a hypoxic environment, contribute to drug resistance. However, in GS-CRT, S-1 may exhibit treatment efficacy even against tumors that are resistant to gemcitabine. Gu et al. reported that PDAC cells rich in CAFs exhibit gemcitabine resistance based on an analysis using a patient-derived xenograft (PDX) model. In the future, if treatment strategies targeting the stroma, such as CAF inhibitors, are developed, it may be possible to enhance the efficacy of gemcitabine while maintaining the GS-CRT protocol. This would lead to improved therapeutic outcomes [57]. Furthermore, in recent years, micro-robots have garnered attention for their small size and diverse propulsion mechanisms, which contribute to efficient movement, targeted guidance and enhanced drug delivery precision within the body [58]. Advances in drug delivery technology for tumors are progressing to overcome the limitations of single-agent therapy. For instance, Lee et al. designed a micro-robot capable of sequentially releasing two different drugs. In their system, gemcitabine (GEM) was bound to the surface of the micro-robot via disulfide bonds, while doxorubicin (DOX) was encapsulated inside the micro-robot for secondary release [59]. By applying similar technologies, more effective future treatment modalities can be developed that would utilize the techniques of combining multiple drugs. For PDAC treatment, this has a potential to significantly improve patients’ survival outcomes.

Furthermore, an international retrospective cohort study (n = 1758) which examined pCR in PDAC patients who underwent resection following pre-operative chemoradiotherapy reported a pCR rate of 4.8% (n = 85). In the same study, the 5-year OS rate was 30% in patients who did not achieve pCR compared to 63% among those who did. Factors which were associated with pCR included pre-operative combination chemotherapy (excluding mFOLFIRINOX, OR 0.48), preoperative conventional radiotherapy (OR 2.03), and pre-operative stereotactic body radiotherapy (OR 8.91) [60].

When we endeavored to identify predictors of DSS among the 49 resected UR-LA cases, preoperative PNI, pathologic venous invasion and postoperative adjuvant chemotherapy were identified in multivariate analysis, as shown in Table 5. Pre-operative nutritional status significantly impacts post-operative outcomes, the feasibility to receive adjuvant therapy and long-term prognosis in pancreatic cancer patients. A systematic review involving 26 studies with a total of 2720 patients concluded that pre-operative malnutrition was associated with an increased risk of post-operative complications and poor long-term prognosis. Specifically, weight loss, low BMI, hypoalbuminemia and a low Prognostic Nutritional Index (PNI) are described as significant risk factors for post-operative outcomes [61]. Additionally, a meta-analysis of 14 studies (3385 patients) on PNI and prognosis after preoperative therapy in PDAC patients also showed that low preoperative PNI was closely associated with worse OS and recurrence-free survival. Although the cut-off values for preoperative PNI in each article varied from 36 to 53.1 and were not consistent, the studies concluded that preoperative PNI may be a promising predictor of prognosis in PDAC patients undergoing radical resection [62]. We previously reported that PNI after CRT was a significant prognostic factor in evaluating inflammatory and nutritional markers and in predicting prognosis after CRT. In that study, a cutoff value of 39 was identified as the dividing point for survival outcomes (MST: 27.2 vs. 15.5 months, *p* = 0.0016) [37]. In the present study, 43.2 was identified as a cut-off value among the UR-LA resected cases, which was similar to our previous report [32]. A report by Kawahara et al. also demonstrated that preoperative PNI was an independent risk factor for the continuation of S-1 adjuvant chemotherapy in patients undergoing surgical resection after neoadjuvant chemotherapy (OR 2.435, *p* = 0.011). For them, 45 was identified as a cutoff value for preoperative PNI and reported lower S-1 completion rates (*p* = 0.02), higher discontinuation rates (*p* = 0.031) and higher rates of grade 2 or higher adverse events from adjuvant chemotherapy (*p* < 0.001) [63]. These findings suggest that preoperative PNI is closely related to adjuvant chemotherapy and may explain the significant difference in survival outcomes observed between patients with and those without adjuvant therapy in the current study, as shown in Figure 6c. Furthermore, we observed that pathological venous invasion had a significant impact on prognosis as there was a marked difference in survival between patients with and without invasion, as shown in Figure 6b. Similarly, Kubo et al. demonstrated that microvenous invasion (MVI) was an independent prognostic factor for OS (HR 2.86, *p* = 0.003) in patients undergoing pancreatectomy after neoadjuvant therapy for R and BR-PDAC. Furthermore, MVI was also reported to be an independent risk factor for liver metastasis (HR 2.38, *p* = 0.016) and multi-site recurrence (HR 1.92, *p* = 0.027) [64]. In the present study, pre-operative CRT may have played an important role in improving survival by reducing microvenous invasion.

Consequently, for patients requiring pancreatectomy for advanced PDAC, it is crucial to maintain a good nutritional status pre-operatively and provide adjuvant therapy post-operatively. For UR-LA-PDAC cases, surgeons should consider performing combined resection and reconstruction of the PV and SMV. In cases with CA and CHA involvement, surgical resection should be considered only if the tumor has sufficiently shrunk after CRT and vascular reconstruction is deemed feasible. The following techniques should also be considered for patients undergoing surgery after preoperative therapy: the artery-first approach [65,66], arterial divestment [66,67], triangle resection [68], and the hanging maneuver [69]. These techniques aim to achieve thorough tumor resection and postoperative preservation of function while avoiding arterial resection. However, cases with extensive tumor infiltration into the SMA generally preclude surgical resection. Therefore, comprehensive evaluation and careful surgical judgment tailored to each individual case are essential.

In recent years, the development of molecular targeted therapies has progressed with a particular focus on KRAS inhibitors. Approximately 90% of PDAC cases harbor KRAS mutations. As a result, the National Cancer Institute (NCI) has identified KRAS targeting as one of the key priorities for pancreatic cancer research over the next decade [70]. Currently, clinical trials for G12C inhibitors such as Sotorasib and Adagrasib are underway and related studies are also actively advancing. Among these inhibitors, pyruvate dehydrogenase kinase (PDK) inhibitors have been suggested to suppress tumor metabolism and inhibit tumor growth in KRAS-mutant PDAC, thereby making them one of the promising areas of continued future research [71]. In particular, adoptive cell therapy (ACT) targeting KRAS mutations represents another key research area in the pursuit of personalized treatment for pancreatic cancer and holds great potential for future applications [72]. Regarding immunotherapy, pembrolizumab (an anti-PD-1 antibody) has been confirmed to be effective in PDAC with MSI-high/dMMR (approximately 1–3%) and the United States’ Food and Drug Administration (FDA) has approved pembrolizumab for metastatic PDAC patients with MSI-high status. In the future, it may be possible to also use pembrolizumab as a personalized treatment for locally advanced pancreatic cancer [73].

This study has several limitations. First, it was a single-center retrospective study. To more accurately evaluate the efficacy of GS-CRT in patients with locally advanced PDAC, a prospective multicenter randomized trial is required. Another minor limitation is the presence of potential confounding factors after GS-CRT and during the postoperative period. For instance, chemotherapy regimens may have been altered following poor responses to GS-CRT or relapse and these may not reflect the most current therapies. Additionally, the pharmacokinetic and pharmacodynamic properties of S-1 are known to differ between Westerner populations and East Asian populations, and the frequency of gastrointestinal side effects (diarrhea) may vary between these population groups. As a result, it remains unclear whether the present findings can directly be applied to Western populations. Furthermore, the definition of tumor resectability used in this study is based on the JPS 8th edition and is not fully consistent with the current NCCN guidelines. Although the two systems are similar, the JPS definitions are entirely based on objective CT evaluations, whereas the NCCN guidelines incorporate subjective judgments such as “safe and complete resection and reconstruction”. Therefore, slight differences may exist in the evaluation of CHA and portal/superior mesenteric vein involvement. Also, the current study mainly focused on the resected cases and failed to explain why survival outcomes were similar among the non-resected patients. Lastly, we did not take any measures to address the risk of selection bias, which could have affected our findings.

## 5. Conclusions

GS-CRT can safely and effectively be used in the treatment of locally advanced PDAC, including advanced cases with UR-LA tumors. Its safety and efficacy is supported by the high treatment completion rate and acceptable levels of adverse events. Among the resected patients, preoperative PS, preoperative CA19-9 levels and histological response were significant prognostic factors of DSS, with adjuvant chemotherapy also playing a crucial role. Moreover, pre-operative nutritional status and post-operative adjuvant chemotherapy were critical in achieving radical surgery in UR-LA patients. These findings underscore the pivotal role of GS-CRT in improving survival and enhancing the potential for surgery in locally advanced PDAC. Therefore, comprehensive pre-operative and post-operative management of PDAC cases are essential to maximize the treatment efficacy and validate GS-CRT as a practical and effective approach within the PDAC treatment strategy.

## Figures and Tables

**Table 1:** Adverse events * during the induction GS-CRT period (n = 351).

	All (n = 351)	R (n = 83)	BR-PV (n = 40)	BR-A (n = 84)	UR-LA (n = 144)
Completion of CRT	347 (98.9%)	80 (96.4%)	39 (97.5%)	84 (100%)	144 (100%)
Grade 3 or higher adverse events	181 (51.6%)	40 (48.2%)	16 (40.0%)	42 (50.0%)	83 (57.6%)
White blood cell decreased	139 (39.6%)	30 (36.1%)	12 (30.0%)	30 (35.7%)	67 (46.5%)
Neutrophil count decreased	113 (32.2%)	24 (28.9%)	6 (15.0%)	29 (34.5%)	54 (37.5%)
Febrile neutropenia	2 (0.6%)	0 (0%)	0 (0%)	1 (1.2%)	1 (0.7%)
Anemia	18 (5.1%)	5 (6.0%)	1 (2.5%)	3 (3.6%)	9 (6.3%)
Platelet count decreased	17 (4.8%)	7 (8.4%)	0 (0%)	4 (4.8%)	6 (4.2%)
Anorexia	13 (3.7%)	3 (3.6%)	1 (2.5%)	5 (6.0%)	4 (2.8%)
Diarrhea	1 (0.3%)	0 (0%)	0 (0%)	0 (0%)	1 (0.7%)
Eczema	0 (0%)	0 (0%)	0 (0%)	0 (0%)	0 (0%)
Peripheral sensoryneuropathy	0 (0%)	0 (0%)	0 (0%)	0 (0%)	0 (0%)

* Adverse events were graded according to Common Terminology Criteria for Adverse Event (CTCAE) version 5.0.

**Table 2:** Clinical characteristics of the patients who underwent GS-CRT.

**(A) All Patients**	**n = 319**
**Before GS-CRT**	
Age	69 (40–87)
Sex (male/female)	193/126
PS (0/1 or 2/3)	305/14
BMI (Kg/m^2^)	21.2 (14.1–37.3)
Hb (g/dL)	13.1 (6.9–17.0)
Alb (g/dL)	3.7 (2.4–4.7)
PNI (prognostic nutrition index)	38.5 (26.2–48.7)
White blood cell counts (/mm^3^)	5760 (2520–13,320)
Neutrophil counts (/mm^3^)	3590 (1150–11,920)
Lymphocyte counts (/mm^3^)	1450 (270–4630)
NLR (neutrophils/lymphocytes ratio)	2.5 (0.6–21.2)
CA19-9 level (U/L)	199.4 (0.1–61,621)
CEA level (ng/mL)	3.7 (0.8–97.3)
Portal venous contact or invasion ≥ 180° (yes/no)	150/169
Celiac axis contact or invasion ≥ 180° (yes/no)	55/264
Superior mesenteric artery contact or invasion ≥ 180° (yes/no)	77/242
Common hepatic artery contact or invasion ≥ 180° (yes/no)	67/252
JPS8th T factor (T1–T3/T4)	121/198
JPS8th N factor (N0/N1a,N1b)	264/55
Tumor size on CT (mm)	32.9 (12.6–90.6)
**(B) Resected Patients**	**n = 184**
**Pre-operative factors**	
Age	67 (40–84)
Sex (male/female)	110/74
PS (0/1 or 2/3)	180/4
BMI (Kg/m^2^)	21.3 (13.6–34.3)
Hb (g/dL)	11.5 (7.6–14.2)
Alb (g/dL)	3.7 (1.7–4.3)
PNI (prognostic nutrition index)	41.0 (22.6–53.0)
White blood cell counts (/mm^3^)	4530 (2300–9930)
Neutrophil counts (/mm^3^)	2880 (900–7880)
Lymphocyte counts (/mm^3^)	900 (316–2120)
NLR (neutrophils/lymphocytes ratio)	3.5 (1.0–11.2)
CA19-9 level (U/L)	28.9 (0.1–1034.3)
CEA level (ng/mL)	3.0 (0.7–41.2)
Portal venous contact or invasion ≥ 180° (yes/no)	67/117
Celiac axis contact or invasion ≥ 180° (yes/no)	17/167
Superior mesenteric artery contact or invasion ≥ 180° (yes/no)	26/158
Common hepatic artery contact or invasion ≥ 180° (yes/no)	28/156
JPS8th T factor (T1–T3/T4)	99/85
JPS8th N factor (N0/N1a,N1b)	171/13
Tumor size on CT (mm)	25.7 (10.4–82.0)
Duration from initial treatment (day)	114 (79–434)
**Intra-operative factors**	
Operative procedures (PD, TP/DP)	152/32
Operation time (minutes)	525 (229–906)
Blood loss (ml)	757 (60–11,937)
Combined resection of portal vein (yes/no)	141/43
Combined resection of common hepatic artery (yes/no)	18/166
Combined resection of celiac axis (yes/no)	6/178
**Histopathological factors**	
Degree of pathological differentiation (well/mod-poor/NE)	88/80/16
Degree of lymphatic invasion (ly0/ly1–3/NE)	158/22/4
Degree of perineural invasion (ne0/ne1–3/NE)	72/108/4
Degree of venous invasion (v0/v1–3/NE)	147/33/4
Degree of the residual tumor (R0/R1–2)	168/16
Degree of histological response (grade1–2/grade 3–4)	116/68
**Post-operative factors**	
Postoperative complications (yes/no) CD > 3	53/131
Adjuvant chemotherapy (yes/no)	155/29
**(C) Resected UR-LA Patients**	**n = 49**
**Pre-operative factors**	
Age	67 (50–76)
Sex (male/female)	29/20
PS (0/1 or 2/3)	48/1
BMI (Kg/m^2^)	21.8 (15.9–34.3)
Hb (g/dL)	11.2 (7.6–14.6)
Alb (g/dL)	3.6 (1.7–4.5)
PNI (prognostic nutrition index)	39.3 (22.6–52.1)
White blood cell counts (/mm^3^)	4510 (2570–9930)
Neutrophil counts (/mm^3^)	2850 (1640–7880)
Lymphocyte counts (/mm^3^)	810 (316–2400)
NLR (neutrophils/lymphocytes ratio)	3.6 (1.5–11.2)
CA19-9 level (U/L)	27.4 (0.7–1034.3)
CEA level (ng/mL)	2.8 (1.4–9.2)
Portal venous contact or invasion ≥ 180° (yes/no)	28/21
Celiac axis contact or invasion ≥ 180° (yes/no)	15/34
Superior mesenteric artery contact or invasion ≥ 180° (yes/no)	23/26
Common hepatic artery contact or invasion ≥ 180° (yes/no)	22/27
JPS8th T factor (T1–T3/T4)	2/47
JPS8th N factor (N0/N1a,N1b)	43/6
Tumor size on CT (mm)	29.7 (14.4–82.0)
Duration from initial treatment (day)	152 (78–489)
**Intra-operative factors**	
Operative procedures (PD, TP/DP)	36/13
Operation time (minutes)	600 (333–801)
Blood loss (mL)	1155 (318–11,937)
Combined resection of portal vein (yes/no)	39/10
Combined resection of common hepatic artery (yes/no)	10/39
Combined resection of celiac axis (yes/no)	5/44
**Histopathological factors**	
Degree of pathological differentiation (well/mod-poor/NE)	22/23/4
Degree of lymphatic invasion (ly0/ly1–3)	43/6
Degree of perineural invasion (ne0/ne1–3)	11/38
Degree of venous invasion (v0/v1–3)	42/7
Degree of the residual tumor (R0/R1)	39/10
Degree of histological response (grade1–2/grade 3–4)	36/13
**Post-operative factors**	
Postoperative complications CD > 3 (yes/no)	13/36
Adjuvant chemotherapy (yes/no)	39/10

**Table 3:** Univariate and multivariate analyses to identify prognostic predictors of disease-specific survival of all re-evaluated patients (n = 319).

	Univariate Analysis			Multivariate Analysis		
Variables Before GS-CRT	HR	95%CI	*p*-Value	HR	95%CI	*p*-Value
Age			0.072			0.385
Sex (male/female)			0.606			
PS (0/1 or 2/3)			0.069	**1.9**	**1.031–3.504**	**0.04**
BMI (Kg/m^2^)			0.423			
Hb (g/bL)	**0.91**	**0.844–0.973**	**0.007**	**0.9**	**0.841–0.971**	**0.006**
Alb (g/dL)			0.316			
PNI (prognostic nutrition index)			0.248			
White blood cell counts (/mm^3^)			0.794			
Neutrophil counts (/mm^3^)			0.802			
Lymphocyte counts (/mm^3^)			0.632			
NLR (neutrophils/lymphocytes ratio)			0.533			
CA19-9 level (U/L)			0.339			
CEA level (ng/mL)			0.095			0.065
Portal venous contact or invasion ≥ 180° (yes/no)	1.327	1.027–1.715	0.03			0.291
Celiac axis contact or invasion ≥ 180° (yes/no)	**1.79**	**1.305–2.451**	**<0.001**	**1.45**	**1.039–2.035**	**0.029**
Superior mesenteric artery contact or invasion ≥ 180° (yes/no)	1.786	1.337–2.384	<0.001			0.09
Common hepatic artery contact or invasion ≥ 180° (yes/no)	1.435	1.063–1.939	0.018			0.28
JPS8th T factor (T1–T3/T4)	**1.94**	**1.468–2.567**	**<0.001**	**1.8**	**1.334–2.416**	**<0.001**
JPS8th N factor (N0/N1a,N1b)			0.804			
Tumor size on CT (mm)	1.012	1.002–1.023	0.015			0.459

**Note:** Bold: variables with *p* < 0.05.

**Table 4:** Univariate and multivariate analyses to identify prognostic predictors of disease-specific survival among all the resected cases (n = 184).

	Univariate Analysis			Multivariate Analysis		
	HR	95%CI	*p*-Value	HR	95%CI	*p*-Value
**Pre-operative factors**						
Age			0.697			
Sex (male/female)			0.373			
PS (0/1 or 2/3)	**5.34**	**1.645–17.359**	**0.005**	**5.34**	**1.612–17.672**	**0.006**
BMI (Kg/m^2^)			0.218			
Hb (g/bL)	0.87	0.769–0.986	0.029			0.346
Alb (g/dL)			0.1			
PNI (prognostic nutrition index)			0.305			
White blood cell counts (/mm^3^)			0.85			
Neutrophil counts (/mm^3^)			0.855			
Lymphocyte counts (/mm^3^)			0.6			
NLR (neutrophils/lymphocytes ratio)			0.308			
CA19-9 level (U/L)	**1**	**1.001–1.004**	**<0.001**	**1**	**1.001–1.003**	**<0.001**
CEA level (ng/mL)			0.733			
Portal venous contact or invasion ≥180° (yes/no)	1.47	1.000–2.171	0.05			0.174
Celiac axis contact or invasion ≥ 180° (yes/no)			0.052			0.406
Superior mesenteric artery contact or invasion ≥ 180° (yes/no)	1.91	1.157–3.136	0.011			0.336
Common hepatic artery contact or invasion ≥ 180° (yes/no)			0.848			
JPS8th T factor (T1–T3/T4)	**1.75**	**1.194–2.567**	**0.004**	**1.89**	**1.275–2.813**	**0.002**
JPS8th N factor (N0/N1a,N1b)			0.907			
Tumor size on CT (mm)	1.02	1.001–1.033	0.033			0.833
Duration from initial treatment (day)			0.292			
**Intra-operative factors**						
Operative procedures (PD, TP/DP)			0.342			
Operation time (minutes)			0.342			
Blood loss (ml)	1	1.000–1.000	0.018			0.641
Combined resection of portal vein (yes/no)			0.243			
Combined resection of common hepatic artery (yes/no)			0.701			
Combined resection of celiac axis (yes/no)			0.122			
**Histopathological factors**						
Degree of pathological differentiation (well/mod-poor/NE)			**0.027**			0.107
Degree of lymphatic invasion (ly0/ly1–3)			0.145			
Degree of perineural invasion (ne0/ne1–3)	1.8	1.197–2.696	0.005			0.073
Degree of venous invasion (v0/v1–3)			0.131			
Degree of the residual tumor (R0/R1)	2.13	1.183–3.835	0.012			0.599
Degree of histological response (grade1–2/grade 3–4)	**1.84**	**1.218–2.779**	**0.004**	**1.7**	**1.084–2.69**	**0.021**
**Post-operative factors**						
Postoperative complications CD > 3 (yes/no)			0.261			
Adjuvant chemotherapy (yes/no)			0.15	**2.25**	**1.204–4.217**	**0.011**

**Note:** Bold: variables with *p* < 0.05.

**Table 5:** Univariate and multivariate analyses to identify prognostic predictors of disease-specific survival among resected cases with unresectable–locally advanced tumors (n = 49).

	Univariate Analysis			Multivariate Analysis		
	HR	95%CI	*p*-Value	HR	95%CI	*p*-Value
**Pre-operative factors**						
Age			0.925			
Sex (male/female)			0.31			
PS (0/1 or 2/3)			0.637			
BMI (Kg/m^2^)			0.305			
Hb (g/bL)			0.061			0.754
Alb (g/dL)			0.147			
PNI (prognostic nutrition index)	**0.94**	**0.892–0.996**	**0.036**	**0.93**	**0.866–0.988**	**0.02**
White blood cell counts (/mm^3^)			0.216			
Neutrophil counts (/mm^3^)			0.86			
Lymphocyte counts (/mm^3^)	0.999	0.998–1.000	0.045			0.161
NLR (neutrophils/lymphocytes ratio)			0.258			
CA19-9 level (U/L)	1.003	1.001–1.006	0.012			0.125
CEA level (ng/mL)	1.172	1.008–1.363	0.039			0.261
Portal venous contact or invasion ≥ 180° (yes/no)			0.786			
Celiac axis contact or invasion ≥ 180° (yes/no)			0.094			
Superior mesenteric artery contact or invasion ≥ 180° (yes/no)			0.309			
Common hepatic artery contact or invasion ≥ 180° (yes/no)			0.26			
JPS8th T factor (T1–T3/T4)			0.104			0.895
JPS8th N factor (N0/N1a,N1b)			0.981			
Tumor size on CT (mm)			0.476			
Duration from initial treatment (day)			0.701			
**Intra-operative factors**						
Operative procedures (PD, TP/DP)			0.355			
Operation time (minutes)			0.373			
Blood loss (ml)			0.1			0.297
Combined resection of portal vein (yes/no)			0.777			
Combined resection of common hepatic artery (yes/no)			0.472			
Combined resection of celiac axis (yes/no)			0.471			
**Histopathological factors**						
Degree of pathological differentiation (well/mod-por/NE)			0.22			
Degree of lymphatic invasion (ly0/ly1–3)			0.527			
Degree of perineural invasion (ne0/ne1–3)			0.168			
Degree of venous invasion (v0/v1–3)	**7.84**	**2.209–27.808**	**0.001**	**8.13**	**2.102–31.408**	**0.002**
Degree of the residual tumor (R0/R1)			0.297			
Degree of histological response(grade1–2/grade 3–4)			0.07			0.239
**Post-operative factors**						
Postoperative complications CD > 3 (yes/no)			0.752			
Adjuvant chemotherapy (yes/no)	**3.43**	**1.349–8.742**	**0.01**	**3.04**	**1.051–8.773**	**0.04**

## Data Availability

The data that support the findings of this study are available from the corresponding author, Mie University, upon reasonable request.

## Supplementary Materials

The following supporting information can be downloaded at: https://www.mdpi.com/article/10.3390/cancers17061048/s1, Figure S1. Disease-specific survival (DSS) curves of GS-CRT PDAC patients according to PS and Hb levels; Table S1. Identifying factors associated with grade 3 or higher adverse events.

## Abbreviations

PDAC	Pancreatic ductal adenocarcinoma
GS-CRT	Gemcitabine plus S-1-based chemoradiotherapy
NAC	Neoadjuvant chemotherapy
DSS	Disease-specific survival
DFS	Disease-free survival
OS	Overall survival
CA	Celiac artery
CHA	Common hepatic artery
SMA	Superior mesenteric artery
SMV	Superior mesenteric vein
PV	Portal vein
CA19-9	Carbohydrate antigen 19-9
CEA	Carcinoembryonic antigen
Hb	Hemoglobin
WBC	White blood cell
PS	Performance status
PNI	Prognostic nutritional index (PNI)
NCCN	National Comprehensive Cancer Network
AHPBA	American Hepatopancreatobiliary Association
MDACC	University of Texas MD Anderson Cancer Center
ECOG	Eastern Cooperative Oncology Group
SSO	Society of Surgical Oncology
R0	Negative resection margins
R1	Positive resection margins
GnP	Gemcitabine/nab-paclitaxel
EUS-FNA	Endoscopic ultrasound-guided fine-needle aspiration
MDCT	Multi-dynamic computed tomography
R	Resecteable
BR-PV	Borderline resectable (superior mesenteric vein/portal vein invasion alone)
BR-A	Borderline resectable (arterial invasion)
UR-LA	Unresectable–locally advanced
JPS	Japan Pancreas Society
CTCAE	Common Terminology Criteria for Adverse Event
MST	Median survival time
NLR	Neutrophils/lymphocytes ratio
PD	Pancreaticoduodenenctomy
DP	Distal pancreatectomy
TP	Total pancreatectomy
CSC	Cancer stem cell
PDX	Patient-derived xenograft
CAFs	Cancer-associated fibroblasts
DOX	Doxorubicin
BMI	Body Mass Index
FDA	Food and Drug Administration
