# Supplementary material for: Impact of Surgical Resection After Induction Gemcitabine Plus S-1-Based Chemoradiotherapy in Patients with Locally Advanced Pancreatic Ductal Adenocarcinoma: A Focus on UR-LA Cases
Source: Cancers (Basel). 2025 Mar 20;17(6):1048. doi: 10.3390/cancers17061048 (PMC11941732; doi:10.3390/cancers17061048)
Supplement: Supplementary file 1 [file cancers-17-01048-s001.zip › cancers-3469706-supplementary.pdf]

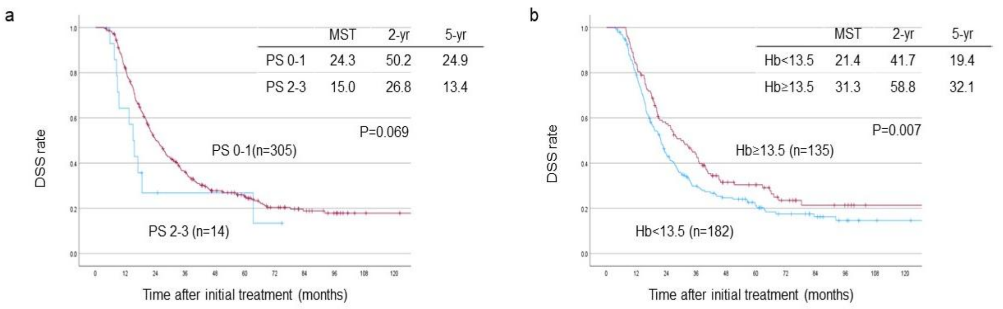


**Figure S1.** Disease specific survival (DSS) curve of GS-CRT PDAC patients according to PS and Hb levels.

There was no significant difference in MST between patients with PS 0-1 (n=305) and those with PS2-3 (n=14) (p=0.069), b) MST in patients with Hb⩾13.5 (n=135) was significantly longer than that in patients with Hb<13.5 (n=182) (MST 31.3M v.s. 21.4, p=0.007). MST: median survival time, PS: performance status.

**Table S1 Identifying factors associated with grade 3 or higher adverse events**

| **Factors** | **n=351** | P-value |
|---|---|---|
| Before GS-CRT | | |
| Age | 69 (40-87) | **0.67** |
| Sex (male/female) | 211/140 | **0.101** |
| PS (0/1 or 2/3) | 334/17 | 0.197 |
| BMI (Kg/m$^2$) | 21.1 (14.1-37.3) | 0.671 |
| Hb (g/dL) | 13.0 (6.9-17.0) | 0.671 |
| Alb (g/dL) | 3.9 (2.5-4.9) | 0.306 |
| PNI (Prognostic nutrition index) | 38.5 (26.2-48.7) | 0.997 |
| White blood cell counts (/mm$^3$) | 5795 (2520-14470) | **<0.001** |
| Neutrophil counts (/mm$^3$) | 3595 (1150-11920) | **<0.001** |
| Lymphocytes counts (/mm$^3$) | 1450 (270-8550) | **0.077** |
| NLR (Neutrophils/lymphocytes ratio) | 2.4 (0.6-21.2) | 0.144 |
| CA19-9 level (U/L) | 207.6 (0.1-61621) | 0.928 |
| CEA level (ng/mL) | 3.7 (0.8-97.3) | 0.601 |
| Resectability(R/BR-PV/BR-A/UR-LA) | 83/40/84/144 | **0.123** |
